# Supplementary material for: BMP3 Affects Cortical and Trabecular Long Bone Development in Mice
Source: Int J Mol Sci. 2022 Jan 12;23(2):785. doi: 10.3390/ijms23020785 (PMC8775420; doi:10.3390/ijms23020785)
Supplement: Supplementary file 1 [file ijms-23-00785-s001.zip › ijms-1523008-supplementary.pdf]

Supplementary materials

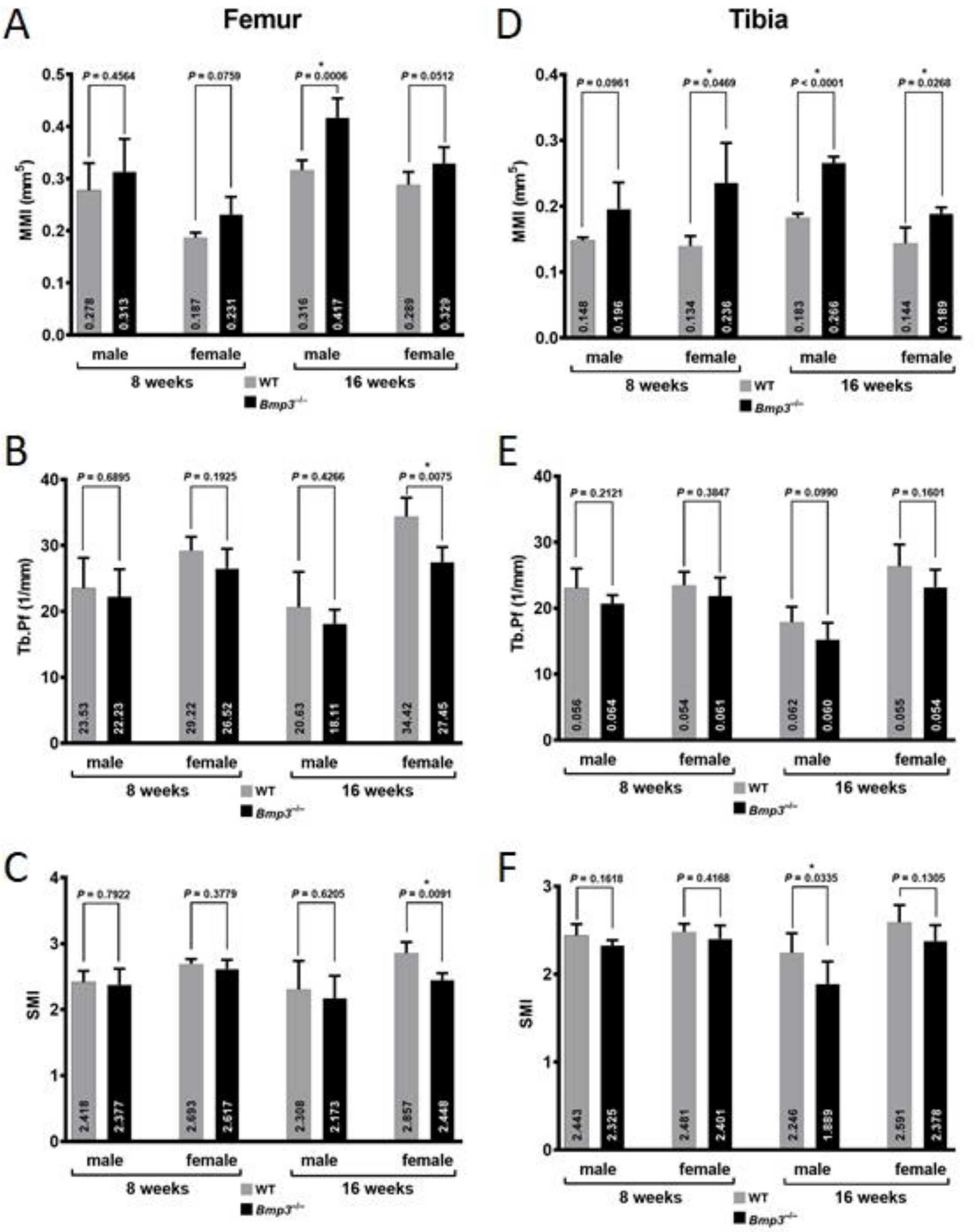

Figure S1. Biomechanical parameters for the cortical and trabecular bone calculated from micro-CT analysis. Femur cortical polar moment of inertia (A). Femoral trabecualr pattern factor (B) and structure model index (C). Tibial polar moment of inertia (D). Tibial trabecula pattern factor (E) and structure model index (F).

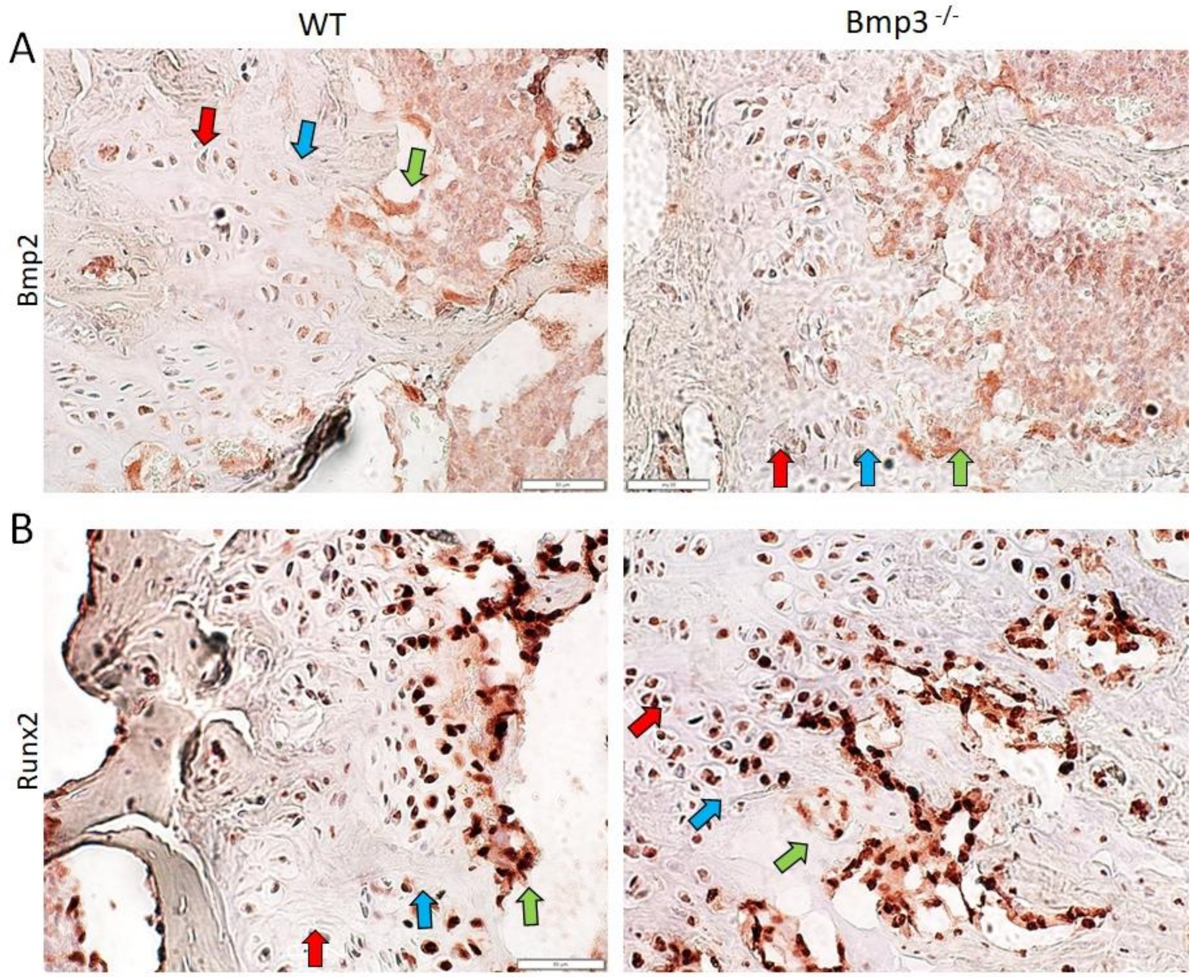

Figure S2. Immunohistochemical analysis of Bmp2 (A) and Runx2 (B) in the femoral distal growth plate in 16-week old male mice. Growth plate layers are depicted by arrows. Red arrows indicate proliferative zone, blue arrow indicate hypertrophic zone, while green arrows indicate primary spongiosa, respectively. Higher expression of Bmp2 can be detected in Bmp3$^{-/-}$ mice. Runx2 in *Bmp3-/-* mice is detected in
